# Recent Progress in Cellulose Nanofibril Hydrogels for Biomedical Applications

**DOI:** 10.3390/polym17172272

**Published:** 2025-08-22

**Authors:** Taeyen Won, MeeiChyn Goh, Chaewon Lim, Jieun Moon, Kyueui Lee, Jaehyeung Park, Kyeongwoon Chung, Younghee Kim, Seonhwa Lee, Hye Jin Hong, Kihak Gwon

**Affiliations:** 1 Department of Biofibers and Biomaterials Science, Kyungpook National University, Daegu 41566, Republic of Korea; wty1196@gmail.com (T.W.); codnjs_1214@naver.com (C.L.); moo1965@naver.com (J.M.); parkj@knu.ac.kr (J.P.); kychung@knu.ac.kr (K.C.); kyh@hespa.or.kr (Y.K.); 2 Institute of Medical Imaging, Hengyang Medical School, University of South China, Hengyang 421009, China; meeichyn1206@gmail.com; 3 Department of Chemistry, Kyungpook National University, Daegu 41566, Republic of Korea; kyueui@knu.ac.kr; 4 KNU G-LAMP Project Group, KNU Institute of Basic Sciences, Kyungpook National University, Daegu 41566, Republic of Korea; 5 Biomedical Research Institute, Kyungpook National University Hospital, Daegu 41940, Republic of Korea; 6 Department of Corporate Support, Healthcare & Spa Industry Promotion Agency, Asan 31442, Republic of Korea; 7 Medical Device Development Center, Osong Medical Innovation Foundation, 123 Osongsaengmyeong-ro, Cheongju 28160, Republic of Korea; lee.seonhwa@kbiohealth.kr; 8 Department of Chemical and Biomolecular Engineering, Yonsei University, Seoul 03722, Republic of Korea

**Keywords:** cellulose nanofibril-based hydrogels, biomaterials, drug delivery, tissue engineering

## Abstract

Cellulose nanofibril (CNF)-based hydrogels, owing to their sustainability, biocompatibility, and versatile mechanical properties, are promising for biomedical applications. This review analyzes the recent advances and biomedical applications of CNF hydrogels. CNF hydrogels can be prepared via physical and chemical crosslinking. Physical crosslinking involves surface charge density control, pH manipulation, and flow-based processing to generate stable networks, whereas chemical crosslinking employs agents such as epichlorohydrin and citric acid to form permanent covalent bonds. These approaches enable precise control over hydrogel properties, including mechanical strength, porosity, and stimuli responsiveness. CNF hydrogels are particularly promising in drug delivery systems and tissue engineering. CNFs as drug delivery vehicles offer enhanced bioavailability and drug loading capacity owing to their open pore structure and large surface area. Recent developments in stimuli-responsive and injectable CNF hydrogels have enabled controlled drug release and improved targeting capabilities. Moreover, CNF hydrogels serve as effective scaffolds for cell growth and tissue regeneration, with applications in cartilage engineering and wound healing. Integrating CNF hydrogels with 3D bioprinting technology has generated complex tissue structures. However, several challenges remain, including the need for the standardization of toxicology assessments, optimization of large-scale production processes, and development of sophisticated control mechanisms for drug delivery. Future research should advance manufacturing technologies, improve long-term stability, and develop standardized testing protocols for regulatory compliance.

## 1. Introduction

**Figure 1:**
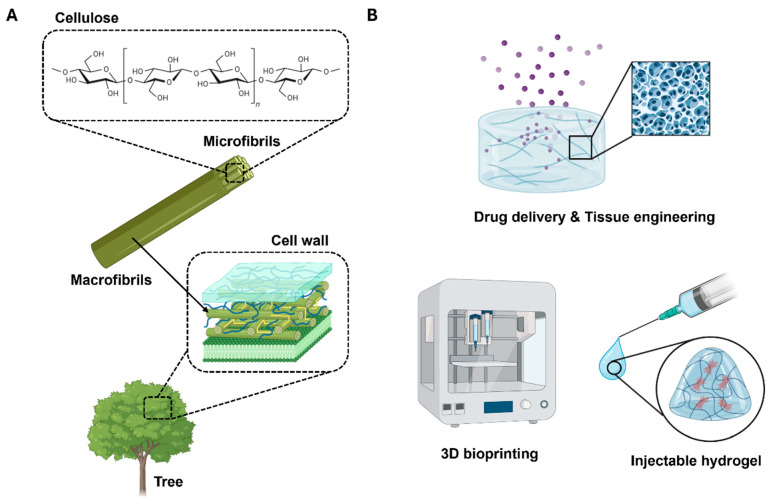
Schematic representation of the (**A**) source of cellulose nanofibrils and the (**B**) application of CNF-based hydrogels (created using Biorender.com).

In recent years, attempts to utilize natural polymers for biomedical applications, including drug delivery, wound dressings, and tissue engineering scaffolds, have notably increased [1,2]. Scientists and industrialists foresee that such attempts could yield environmentally friendly products that are renewable and sustainable. Among various natural polymers, cellulose has garnered significant attention in the biomedical field owing to its biocompatibility, structural tunability, and natural abundance, rendering it ideal for applications such as drug delivery, wound dressing, and tissue engineering (Figure 1A).

The advent of nanotechnology suggests new means for cellulose utilization, particularly in the form of nanocellulose. Nanocellulose, encompassing both cellulose nanocrystals (CNCs) and cellulose nanofibrils (CNFs), has garnered substantial attention from academic and industrial researchers owing to its versatile properties and extensive applications [3,4]. CNFs, occasionally designated as nanofibrillated cellulose or microfibrillated cellulose, are malleable, elongated fibrils with lengths exceeding 1 μm and a cross-section of approximately 5 nm. Typically, CNFs are produced by the high-energy mechanical homogenization of wood pulp, often in conjunction with enzymatic treatment [5], 2,2,6,6-tetramethyl-1-piperidinyloxy (TEMPO) oxidation [6], or other chemical modifications [7], to enhance colloidal stability and reduce the energy input required for fibrillation.

The distinctive structural and physicochemical characteristics of CNFs render them particularly well suited for biomedical applications. CNFs exhibit exceptional physical and chemical properties, including high tensile strength and modulus (in the range of 130–150 GPa), a large specific surface area (up to several hundred meters square per gram), low density (1.6 g/cm^3^), reactive surface chemistry, and intrinsic biodegradability and renewability [8]. These attributes, in conjunction with a high aspect ratio and partially crystalline nature, enable CNFs to form robust networks at low concentrations (<1 wt%) [9]. Interestingly, the usability of CNFs can be extended further when combined with a hydrogel system. Hydrogels represent a class of soft materials formed by three-dimensional networks of crosslinked polymer chains that can hold large volumes of water (up to 99.9%). The combination of CNFs with hydrogels yields an innovative biomaterial that synergizes the inherent properties of CNFs with the advantages of hydrogels.

CNF hydrogels have attracted considerable attention owing to their superior biocompatibility and biodegradability, rendering them suitable for safe application in biomedical and tissue engineering [10,11]. The versatility of CNF hydrogels permits their adaptation to specific biomedical applications, each of which has a unique set of requirements (Figure 1B). For example, cell and organoid cultures necessitate a malleable three-dimensional mechanical support coated with adhesion proteins that exhibit a sol–gel transition, thereby allowing the biological material to be manipulated at various stages [12]. In tissue engineering applications, such as engineered cartilage and skin regeneration, CNF hydrogels can be designed to control mechanical properties, including strength, rigidity, and elongation, and facilitate processing into complex shapes [13,14]. Moreover, in the field of drug delivery, CNF hydrogels have the potential to safeguard therapeutic agents until the target site is reached, where the agents are released in a controlled manner [15]. In diagnostic applications, these hydrogels can be designed to selectively retain a specific marker or biomolecule at a very low concentration and communicate the presence or concentration of this analyte [16].

CNF-based hydrogels offer notable advantages in terms of biocompatibility, renewability, and mechanical tunability. However, the widespread application of these hydrogels in the biomedical field has been constrained by several technical and chemical limitations. In particular, conventional fabrication methods often require the use of toxic polyfunctional crosslinkers that can leave harmful residues and require time-consuming purification. These drawbacks raise concerns regarding biosafety and hinder scalable production [17]. To address these limitations, novel crosslinking strategies that exploit the inherent functional groups of CNFs must be developed, enabling the design of safe, sustainable hydrogel systems.

This review provides comprehensive information regarding the fabrication and characterization methods used for CNF-based hydrogels and presents case studies of the use of these hydrogels in various biomedical applications, including drug delivery and tissue engineering. Furthermore, the current technical challenges and prospective research directions are examined. This review elucidates the potential of CNF hydrogels and clarifies their contribution to future biomedical research and industrial development.

## 2. Methods for Preparing CNF Hydrogels

### 2.1. Physical Crosslinking Methods

**Figure 2:**
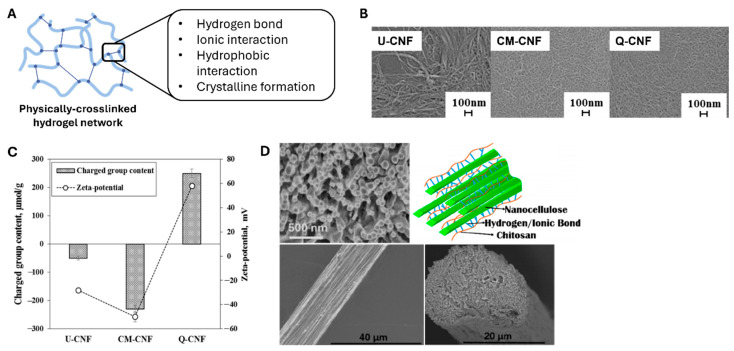
Preparation of CNF hydrogels via physical crosslinking methods. (**A**) Exemplary interactions that construct physically crosslinked hydrogel networks (created using Biorender.com). (**B**) Scanning electron microscope (SEM) images of untreated (U-CNF), carboxymethylated (CM-CNF), and quaternized (Q-CNF) CNFs. (Reproduced/Adapted with permission from [21], MDPI, 2021.) (**C**) Zeta potentials of U-CNF, CM-CNF, and Q-CNF. (Reproduced/Adapted with permission from [21], MDPI, 2021). (**D**) SEM images of the aligned nanocellulose-based filaments generated via flow-assisted assembly. (Reproduced/Adapted with permission from [22], American Chemical Society, 2020.)

CNF hydrogels formed via physical crosslinking exhibit distinct structural and rheological characteristics, primarily owing to the anisotropic morphologies and high aspect ratios of nanocellulose fibrils. Unlike conventional polymer-based hydrogels that typically originate from molecular solutions, CNF dispersions exist as colloidal suspensions, critically influencing gelation mechanisms and viscoelastic behavior [18,19]. Fibrils, measuring between 5 and 50 nm in diameter and extending up to several microns in length, can entangle and form percolated networks via hydrogen bonding and van der Waals interactions, enabling stable hydrogel formation without the presence of chemical crosslinkers [18,20]. Figure 2A reveals that the physical crosslinking of such polymeric networks involves conjugation between polymeric chains via reversible interactions such as hydrogen bonding, ionic interactions, hydrophobic interactions, and crystalline formation.

CNF hydrogels can be crosslinked through several key approaches, and the most fundamental approach involves controlling surface charge density. At a given solid content, increasing the surface charge density of a CNF prevents agglomeration by electrostatic repulsion and promotes network entanglement. Im et al. [21] reported that three different composite hydrogels of CNFs and polyvinylpyrrolidone (PVP) were prepared with various surface charges of CNFs (Figure 2B,C). The group containing untreated CNFs (U-CNFs) possessed a zeta potential value of 0 mV. The hydrogels with carboxymethylated (CM-CNF) and quaternized (Q-CNF) CNFs possessed zeta potentials of −40 mV and +70 mV, respectively (Figure 2C). Interestingly, these three hydrogels demonstrated different transmittance values, crystallinities, distributions of nanofibrils, shear viscosities, and storage moduli.

Hydrogel gelation is highly dependent on controlling pH and ionic strength. Adding salt or reducing solution pH induces gelation by reducing surface charge via counterion-driven charge screening of surface carboxyl groups. The stability of these gels strongly depends on pH and salt concentration, which directly affect the network-bound water content and overall gel structure [23]. Fall et al. demonstrated that the degree of deprotonation and the number of charged carboxyl groups in hydrogels can be precisely controlled by varying salt concentration and pH [23]. Moreover, CNF concentration influences hydrogel formation, and studies have shown that stable gels can form at concentrations as low as 0.125 wt% when using enzymatically treated and homogenized CNFs [5]. This is particularly significant as it represents a concentration two orders of magnitude lower than that required for other nanocellulose materials [24].

Furthermore, structured CNF-based hydrogels are being developed by controlling the orientation of CNFs. Håkansson et al. [25] demonstrated that hydrodynamic forces and ionic interactions can be used to achieve fibril alignment and gelation by exposing CNFs to a focused flow. Similarly, Cai et al. [22] fabricated aligned filaments solely consisting of CNFs via flow-assisted assembling, as illustrated in Figure 2D. The aligned structure imparted better mechanical properties to the CNF-based hydrogel than the randomly oriented nanofibrils and improved applicability in the biomedical engineering field, such as muscle and neuronal engineering. Moreover, the crosslinking density strengthens the overall mechanical modulus of the hydrogel. However, it is also critical to consider the viscoelasticity of the CNF-based hydrogel resulting from the crosslinking density since the modulus exceeding a certain extent without degradability possibly hinders migration and proliferation of cells embedded within the network.

### 2.2. Chemical Crosslinking Methods

**Figure 3:**
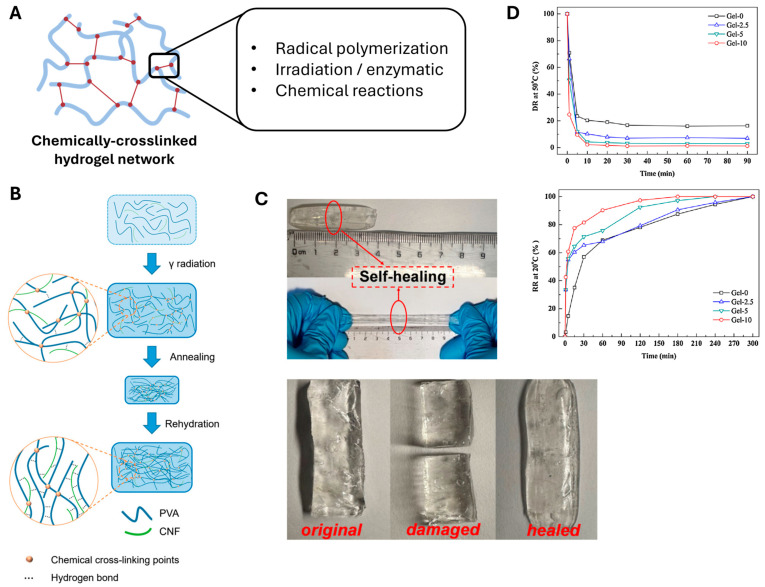
Preparation of CNF hydrogels via chemical crosslinking. (**A**) Exemplary interactions that yield chemically crosslinked hydrogel networks (created using Biorender.com). (**B**) Schematic representation of chemically conjugated network within PVA/CNF hydrogel upon γ-irradiation. (Reproduced/Adapted with permission from [35], MDPI, 2024). (**C**) Self-healing features of a CNF/PVA hydrogel demonstrated by observing full healing after being cut in half (Reproduced/Adapted with permission from [36], American Chemical Society, 2024). (**D**) Difference in deswelling (top) and swelling kinetics (bottom) of PNIPAm-CNF hydrogel due to changes in CNF content (0, 2.5, 5, 10%). (Reproduced/Adapted with permission from [37], Elsevier, 2016).

Chemically crosslinking CNFs creates strong and permanent network structures via various bonding mechanisms (Figure 3A). Chemically crosslinked networks can be achieved via covalent bonds formed through radical polymerization, chemical reactions, irradiation, or enzymatic reactions [26,27,28]. The preparation process begins with the challenging step of dissolving cellulose chains, governed by the balance between entropy and molecular interactions [29,30]. To commence the gelation procedure, different chemical procedures and modification protocols have been developed to dissolve cellulose in water or organic solvents [31,32]. For instance, aqueous solutions of NaOH/urea and LiOH/urea have been used to dissolve cellulose at temperatures as low as −10 °C [33,34].

Radical polymerization can also induce chemical crosslinking of CNF-based hydrogel, as demonstrated by Chen et al. The group demonstrated the fabrication of a composite hydrogel consisting of poly(vinyl alcohol) (PVA) and CNF through γ-irradiation, as shown in Figure 3B [35]. When CNF components are irradiated with γ-rays, free radicals are generated, which then form stable chemical bonds with hydrogen atoms in PVA components. Specifically, hydrogen atoms of the hydroxyl group and methylene group in both CNF and PVA form tertiary and secondary radicals, respectively, upon irradiation. These radicals eventually lead to formation of a 3D network to fabricate a hydrogel network. Basically, in this case, CNF acts as a crosslinker, and the composite form of natural polymer (CNF) and synthetic polymer (PVA) strengthen the mechanical properties of the final PVA/CNF hydrogel.

Composite hydrogel preparation offers another approach to enhance mechanical properties. CNFs can be used as reinforcing agents in polymer matrices; however, the loading levels of these CNFs must be carefully controlled to avoid entanglement. Such composites are more commonly prepared using synthetic polymers, such as PVA, poly (ethylene glycol) (PEG), or polyacrylamide, because such polymers are stable and malleable with excellent mechanical properties. Zhang et al. [36] and Takeno et al. [37] successfully fabricated a CNF/PVA hydrogel crosslinked with borax. They demonstrated that the mechanical properties of the PVA-based hydrogel improved owing to the addition of CNF and borax. In particular, Takeno et al. [37] emphasized the effect of CNF size on gel properties. The composite gel containing the CNF with a smaller length demonstrated better stretchability than the ones with longer fiber lengths. The self-healing capability of these two reported composite hydrogels was another significant feature. As shown in Figure 3C, the CNF/PVA hydrogel cut in half was healed in 5 min of physical contact.

The self-healing capacity of these composite CNF/PVA hydrogels reveals that chemical crosslinking can impart various interesting functionalities to the system. For example, ultraviolet radical polymerization has been used to prepare hydrogels from bacterial nanocellulose and poly(2-hydroxyethyl methacrylate). These composite hydrogels exhibit significantly improved tensile strengths and Young’s moduli with respect to single-component systems [38]. Moreover, temperature-responsive properties can be incorporated via specific chemical modifications. Specifically, poly(N-isopropylacrylamide) (PNIPAm)-based CNF hydrogels have demonstrated characteristic temperature-dependent swelling behavior (Figure 3D). Therefore, the mechanical properties of these thermo-responsive systems can be tuned by controlling the CNF content [37,39].

## 3. Biomedical Applications of CNF Hydrogels

### 3.1. Drug Delivery Systems

Drug delivery systems represent bioengineered technologies designed for the targeted transport of therapeutic agents to specific tissues and organs. These systems incorporate carrier vessels and coating treatments to control the release of medicines and biomolecules [40]. Such modifications enhance pharmacokinetics and optimize the biodistribution of substances in the human body. The development of effective drug delivery systems requires careful consideration of stability factors, including pH, ionic strength, and temperature variations before reaching the target site, to prevent premature release and ensure controlled release [41]. CNF-based hydrogels have emerged as promising carriers for bioactive molecules owing to their unique advantages involving nanostructures, biocompatibility, biodegradability, and tunable surface chemistry [42,43,44]. By incorporating functional groups or additional components, CNF hydrogels can be engineered to achieve stimuli-responsive release, sustained and sequential drug delivery, or injectable and localized therapeutic administration.

#### 3.1.1. Stimuli-Responsive Hydrogels

The development of stimuli-responsive CNF hydrogels has garnered particular attention due to their ability to release drugs on demand in response to specific stimuli such as pH, temperature, mechanical force, light, and ionic strength. These systems offer precise spatiotemporal control over therapeutic delivery, making them attractive candidates for next-generation biomedical applications.

**Figure 4:**
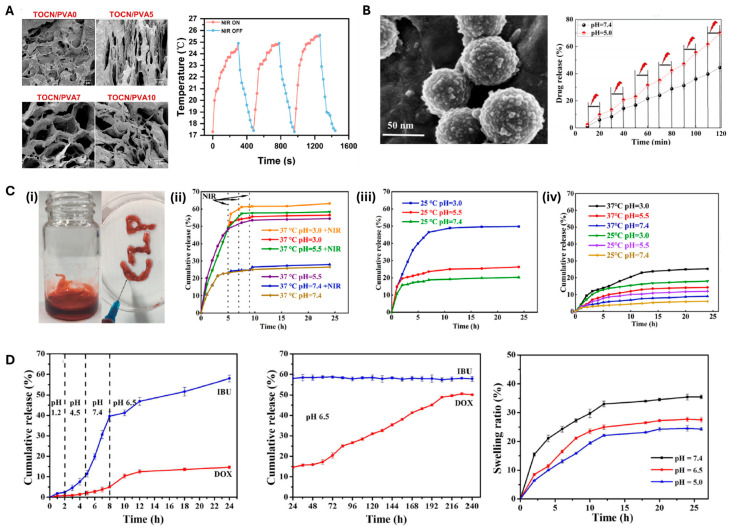
Application of CNF-based hydrogels in drug delivery systems. (**A**) SEM images for the TOCN/PVA hydrogel with changes in PVA content (**left**), and the NIR-responsive temperature changes observed in the hydrogel system (**right**). (Reproduced/Adapted with permission from [47], Elsevier, 2024). (**B**) SEM images of tetracycline hydrochloride (TH) drug-loaded mesoporous polydopamine (MPDA) particles wrapped in graphene oxide sheets (**left**), and release profile for TH from MPDA-encapsulated CNF hydrogel at different pH values (**right**). (Reproduced/Adapted with permission from [51], American Chemical Society, 2020). (**C**) Characterization of the tri-stimuli-responsive injectable hydrogel (CNF-TRIH@DOX): (**i**) Photographic image of the hydrogel and its injectability, the release profile of the loaded DOX depending on the hydrogel responsiveness to (**ii**) NIR, (**iii**) pH, and (**iv**) temperature. (Reproduced/Adapted with permission from [54], Elsevier, 2022.) (**D**) Cumulative release of drugs (IBU and DOX) from CNF/SA hydrogel in response to different pH values, and the swelling ratio of the hydrogel changing at different pH (5.0, 6.5, and 7.4). (Reproduced/Adapted with permission from [55], Elsevier, 2025).

Masruchin et al. [45] synthesized dual-responsive composite hydrogels based on TEMPO-oxidized CNFs and thermally responsive PNIPAm for drug delivery. These hydrogels responded to both pH and temperature. The pH sensitivity of these hydrogels originates from the tunable ionization of the carboxyl groups on CNFs, whereas temperature responsiveness is imparted by PNIPAm, enabling control over swelling behavior via external thermal input. In another significant development, Zhang et al. [46] fabricated pH-responsive gel macrospheres using sodium alginate (SA) and TEMPO-oxidized CNFs for probiotic delivery. These macrospheres remained stable in simulated gastric fluid (SGF) due to reduced electrostatic repulsion and increased hydrogen bonding in acidic conditions and subsequently swelled in simulated intestinal fluid (SIF) to release their payload. The addition of CNFs significantly enhanced the mechanical strength and structural integrity of the macrospheres, improving resistance to shrinkage and disintegration under gastrointestinal conditions. Further advancing CNF-based intelligent hydrogels, Zhang et al. [47] developed a dual-stimuli responsive system composed of polyvinyl alcohol (PVA), polydopamine (PDA), and two-step oxidized CNFs (TOCN) for combined chemo-photothermal breast cancer therapy. TOCN introduced abundant carboxyl groups that enhanced doxorubicin (DOX) loading via electrostatic and hydrogen bonding interactions. The system exhibited controlled DOX release under mildly acidic (pH 6.8) and elevated temperature (42 °C) conditions. Additionally, PDA enabled near-infrared (NIR) photothermal responsiveness, allowing precise on-demand drug release and enhanced therapeutic efficacy (Figure 4A).

In addition to pH- and temperature-responsive designs, CNF-based hydrogels have also been explored for light-triggered controlled drug release. Notably, Lem et al. developed a far-red light-responsive platform using an anionic nanofibrillated cellulose (ANFC) hydrogel loaded with both a tetra-cationic zinc phthalocyanine photosensitizer (ZnPc(MePy)4) and cellulose-binding liposomes [48]. Upon irradiation at 730 nm, an optimal wavelength for deep tissue penetration, the photosensitizer generated singlet oxygen, which oxidatively disrupted the liposomal membranes and triggered the release of encapsulated hydrophilic drugs [48]. The ANFC hydrogel acted as a stable, biocompatible reservoir that confined ROS activity within the matrix, ensuring spatially controlled drug release.

Beyond chemical and photothermal responsiveness, mechanical stimuli have also been harnessed for smart release. Park et al. developed a mechanically responsive semi-interpenetrating polymer network (semi-IPN) hydrogel composed of TEMPO-oxidized bacterial cellulose nanofibers (BCNFs) and polyacrylamide (PAM) [49]. By tuning the aspect ratio of BCNFs, the hydrogel’s mechanical properties, including compressive strength and elasticity, were significantly enhanced. The incorporation of BCNFs reduced hydrogel permeability, slowing passive drug diffusion while enabling on-demand release upon mechanical compression. Notably, drug diffusion was selectively enhanced in hydrogels with higher BCNF content under compressive stimulation, without compromising structural integrity under repeated mechanical stress. This system offers a promising platform for wearable, pressure-sensitive drug delivery applications, such as artificial skin patches.

Together, these studies underscore the versatility of CNF-based hydrogels in engineering multi-responsive drug delivery platforms, capable of reacting to physiological and external cues for precision-controlled therapeutic outcomes.

#### 3.1.2. Composite and Nanocomposite Hydrogels

A major limitation of conventional hydrogels in drug delivery applications is their tendency to exhibit an initial burst release and relatively low drug loading capacity [50]. To address these challenges, composite and nanocomposite hydrogels have been developed to enhance structural integrity, regulate release kinetics, and improve drug encapsulation efficiency. These improvements are typically achieved by either incorporating functional nanoparticles into CNF-based hydrogel matrices or embedding CNFs into other polymer systems to reinforce the hydrogel network. Such hybrid systems integrate the unique properties of both CNFs and functional additives, offering synergistic advantages that make them highly suitable for sustained and targeted drug delivery.

Liu et al. [51] constructed CNF-based composite hydrogels (MDPA@GO/CNF) by incorporating mesoporous polydopamine (MPDA) and graphene oxide (GO) to achieve sustained drug release and safe delivery of tetracycline hydrochloride (TH). MPDA loaded with TH was wrapped in GO sheets and embedded into CNF-based hydrogel (Figure 4B, left). In this system, CNFs serve as a hydrophilic fibrous matrix, offering abundant hydroxyl and carboxyl groups that promote structural integrity and crosslinking through non-covalent interactions with GO and calcium ions. The incorporation of MPDA@GO into the CNF matrix successfully enhanced the sustained and controlled release of TH compared to pure CNF hydrogel. Furthermore, drug release from the composite hydrogel could be regulated by environmental pH and near-infrared (NIR) irradiation (Figure 4B, right).

In another study, Yunnan Baiyao (YNBY) particles were incorporated into a CNF-based composite hydrogel to construct a wearable patch for sustained transdermal drug delivery [52]. In this formulation, CNFs form a highly entangled fibrous network that reinforces the mechanical strength of the hydrogel and reduces its swelling rate by increasing crosslinking density. This network acts as a diffusion barrier, indirectly modulating the release of embedded drug molecules. The incorporated YNBY serves as a particulate drug reservoir, gradually releasing its bioactive components, such as Panax notoginseng saponin R1, through a combination of diffusion and matrix swelling. The interaction between YNBY and the CNF hydrogel matrix further contributes to the sustained and controlled drug release behavior. Thus, this CNF-based nanocomposite hydrogel presents a promising strategy for long-lasting transdermal therapeutic applications.

Similarly, CNF/Zinc oxide nanohybrids (ZONHs) were incorporated into a hydrogel composed of starch, gelatin, and itaconic acid to create a pH-sensitive nanocomposite system for sustained drug delivery [53]. In this formulation, CNFs facilitate the stable dispersion of ZnO nanoparticles, ensuring uniform distribution and consistent functionality throughout the hydrogel matrix. The resulting ZONHs serve as reinforcing agents, enhancing the hydrogel’s water-swelling capacity and regulating drug release through strong interfacial interactions with the polymer network and encapsulated drug. Moreover, at higher pH levels, the hydrogel demonstrates increased swelling, promoting more effective diffusion of the encapsulated drug. Thus, this CNF-based nanocomposite system offers promising potential as a sustainable drug delivery platform in biomedical applications.

Composite and nanocomposite hydrogels incorporating cellulose nanofibers (CNFs) provide enhanced structural and functional properties for drug delivery systems. Through integration with various functional additives, these hydrogels offer improved mechanical strength, swelling control, and sustained drug release. Their versatility makes them suitable for a broad range of biomedical applications.

#### 3.1.3. Injectable and Localized CNF Hydrogels

Injectable hydrogels have emerged as highly promising platforms for localized drug delivery due to their three-dimensional carrier capacity, biocompatibility, minimally invasive administration, and ability to conform to irregular tissue shapes. In recent years, they have been extensively studied for in vivo drug delivery, particularly in tumor therapy [56]. Cellulose nanofibrils (CNFs), owing to their inherent pseudoplastic and thixotropic behavior, can form shear-thinning hydrogels suitable for injection. These properties enable CNF hydrogels to be easily administered and to rapidly recover their structure post-injection, making them ideal candidates for localized therapeutic applications. For instance, Lauren et al. [57] developed technetium-99m-labeled CNF hydrogels to enable real-time tracking of hydrogel localization and drug release in vivo. The hydrogel exhibited sustained release of large molecules, making it particularly suitable for long-term or depot-based delivery, especially for biologics and protein therapeutics. These biocompatible and non-toxic CNF hydrogels were enzymatically degradable into glucose via localized cellulase activity, enabling an additional level of spatial and temporal control. Such systems demonstrate the potential of CNF-based injectable hydrogels for precision drug delivery in dynamic biological environments.

As shown in Figure 4C, Chen et al. developed a tri-stimuli-responsive injectable hydrogel (CNF-TRIH@DOX) based on TEMPO-oxidized cellulose nanofibrils (CNFs), modified with polyethyleneimine (PEI) and poly(N-isopropylacrylamide) (PNIPAm) to confer responsiveness to near-infrared (NIR) light (Figure 4C(ii)), pH (Figure 4C(iii)), and temperature (Figure 4C(iv)) for tumor therapy [54]. Combined with alginate and chitosan, CNF formed a 3D nanocage-like hydrogel structure with high loading capacity for doxorubicin (DOX) and Prussian blue nanoparticles (PBNPs), the latter acting as a contrast agent for imaging via electrostatic interactions. Thus, CNF plays a central role not only as a structural backbone and responsive switch for stimuli-triggered release, but also as a functional component that enables theranostic capabilities through intelligent drug delivery and imaging performance.

In summary, CNF-based injectable hydrogels represent a versatile and promising platform for localized and controlled drug delivery. Their unique rheological properties, biocompatibility, and ability to form responsive networks enable precise spatial and temporal control over therapeutic release. By integrating functional modifications and responsive components, CNF hydrogels can be tailored for advanced biomedical applications such as tumor-targeted therapy and real-time imaging. These systems exemplify the potential of smart, minimally invasive drug delivery technologies in addressing complex clinical challenges.

#### 3.1.4. Sustained and Sequential Release Hydrogels

Despite these advances, several challenges remain in the development of CNF-based drug delivery systems. A key area for improvement is the design of hydrogel systems capable of simultaneously releasing multiple drugs at controlled, varying rates, an approach that is particularly advantageous for complex diseases like cancer [58].

For instance, a recent study demonstrated a sequential drug delivery system (SDS) using a cellulose nanofiber/sodium alginate (CNF/SA) hydrogel embedded with doxorubicin-loaded calcium carbonate hollow microspheres (CaCO_3_/DOX HMs) and ibuprofen (IBU) for colon cancer treatment [55]. In this system, CNF plays a crucial role in forming a pH-sensitive hydrogel matrix that enables stepwise drug release. At intestinal pH (~7.4), carboxyl group deprotonation in CNF and SA leads to electrostatic repulsion and matrix swelling, allowing the release of IBU to relieve pain. As the pH drops to ~6.5 in the colon, decomposition of the CaCO_3_ microspheres triggers the release of DOX for localized chemotherapy (Figure 4D). Thus, CNF contributes to both structural support and stimuli-responsive behavior, enabling targeted and temporally controlled release of multiple therapeutics.

CNF-based hydrogels offer significant promise as intelligent drug delivery platforms, particularly for localized and combination therapies. Their tunable chemical structure, responsiveness to physiological stimuli, and compatibility with other biomaterials make them ideal candidates for advanced delivery systems. As demonstrated by recent sequential delivery strategies, CNF enables precise spatial and temporal control over multi-drug release, addressing the complex needs of diseases like cancer. Moving forward, further research into multi-responsive and multifunctional CNF-based hydrogels will be key to unlocking their full clinical potential in personalized and effective treatment strategies.

### 3.2. Tissue Engineering

The fundamental requirements for tissue engineering scaffolds include the ability to promote nutritional transport, vascularization through their porous gel structure [57,59,60], and natural degradation in body fluids to eliminate the need for surgical removal [59]. In addition, scaffolds must exhibit sufficient mechanical strength to support cell adhesion, proliferation, and differentiation into specialized, functional tissues.

#### 3.2.1. Scaffold Materials

In recent years, CNF-based hydrogels have garnered considerable attention in tissue engineering applications due to their highly hydrated three-dimensional porous structure that mimics biological tissue [42,61,62]. Moreover, CNFs serve as effective mechanical reinforcement agents, enhancing the structural integrity of hydrogel scaffolds without compromising biocompatibility [58,63]. CNF-based scaffolds have been explored for a variety of tissue engineering applications, including cartilage, meniscus, bone, intervertebral disc, cardiac, corneal, neural, and tracheal regeneration (Table 1).

**Figure 5:**
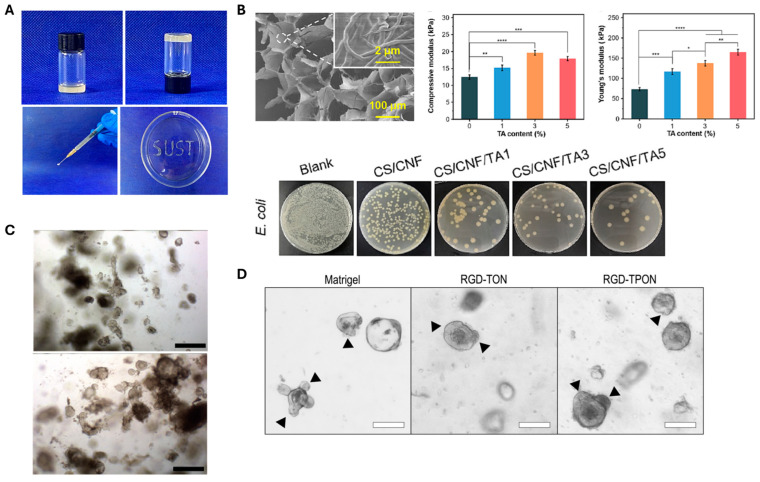
Application of CNF-based hydrogels in tissue engineering. (**A**) Demonstration of sol–gel state and injectability of CNF-based hydrogel in composite with oxidized alginate and gelatin. (Reproduced/Adapted with permission from [65], Elsevier, 20203). (**B**) SEM images and modulus of TA-incorporated CNF hydrogel (**top**) and the antibacterial activity of the CNF/TA hydrogel (**bottom**). *: *p* < 0.05, **: *p* < 0.01, ***: *p* < 0.001, ****: *p* < 0.0001). (Reproduced/Adapted with permission from [77], Elsevier, 2024). (**C**) Maturation of liver organoids within TEMPO-oxidized CNF hydrogels at day 1 (**top**) and day 7 (**bottom**) of encapsulation. Scale bar = 500 μm. (Reproduced/Adapted with permission from [78], Wiley, 2020). (**D**) The morphology of intestinal organoids with buddings (indicated with black arrows) compared after 4 days of culture within Matrigel, RGD-TON, and RGD-TPON. Scale bar = 100 μm. (Reproduced/Adapted with permission from [79], American Chemical Society, 2021).

In the case of bone regeneration, one of the major limitations of natural polymer-based hydrogels is their insufficient mechanical strength, which hinders their effectiveness in load-bearing bone repair applications. To overcome this challenge, Cui et al. developed an injectable and self-healing semi-interpenetrating network (semi-IPN) hydrogel composed of oxidized alginate (OSA), gelatin (Gel), and CNFs, using a one-step synthesis method without the need for external crosslinking agents [65]. In this system, OSA served as a natural crosslinker by forming dynamic imine bonds with the amino groups of gelatin via Schiff base reactions. CNFs contributed to the formation of the hydrogel network through physical interactions, primarily hydrogen bonding, while also acting as mechanical reinforcement agents, thickeners, and rheological modifiers. The mechanical strength of the hydrogel increased with higher CNF content, and the formulation exhibited shear-thinning behavior, facilitating injectability (Figure 5A). Interestingly, while CNFs improved the structural integrity, they also reduced viscosity, which is advantageous for syringe-based administration. Functionally, the OSA/Gel/CNF hydrogel demonstrated excellent biomineralization capability, attributed to the abundant carboxyl and hydroxyl groups on the CNFs, which created strong negative dipoles for chelating free calcium ions. Furthermore, the hydrogel promoted MC3T3-E1 preosteoblast cell viability, proliferation, and osteogenic differentiation, highlighting its potential as an effective injectable scaffold for bone tissue engineering.

Injectable CNF-based hydrogels are highly attractive for minimally invasive applications due to their ability to conform to irregular defect geometries [76]. In addition to bone repair, they have been applied in intervertebral disc regeneration, where both mechanical reinforcement and injectability are essential. Doench et al. [67] developed an injectable CNF-filled chitosan hydrogel with tunable rheological and mechanical properties. CNFs were incorporated without compromising injectability, as their alignment under high shear minimized flow resistance. Rheological studies showed that CNFs formed hydrogen bonding and hydrophobic interactions with CHI, increasing viscosity at low shear rates and improving post-injection stability. The addition of CNFs also enhanced the elastic modulus, mimicking native disc stiffness. In vivo pig studies demonstrated effective implant localization, restoration of disc viscoelasticity, and increased disc height. These findings highlight the potential of CNF/CHI hydrogels as non-cellularized, bioactive materials for disc repair.

In cardiac tissue engineering, Hou et al. prepared a conductive CNF-based hydrogel system by polymerizing polypyrrole (PPy) within a TEMPO-oxidized CNF (TOCN) matrix [72]. The carboxyl groups introduced through TEMPO oxidation enabled hydrogel formation via ionic crosslinking with ferric ions (Fe^3+^), resulting in a stable and interconnected network. Subsequent in situ polymerization of PPy within the TOCN matrix not only imparted electrical conductivity but also mitigated aggregation issues, thereby preserving the mechanical integrity of the hydrogel. The resulting TOCN–PPy composite hydrogel exhibited both suitable mechanical strength and electrical conductivity, effectively mimicking the native properties of myocardial tissue. Notably, cardiomyocytes cultured on the scaffold showed enhanced expression of myocardial-specific proteins, including connexin 43 and cardiac troponin T, indicating favorable cell–material interactions and support for cardiac phenotype maintenance. These findings highlight the potential of TOCN–PPy composite hydrogels as promising scaffolds for myocardial tissue regeneration.

Overall, CNF-based hydrogels are versatile scaffold materials with high water content, ECM-like structure, and strong mechanical reinforcement capabilities. They enhance the strength, stability, and biofunctionality of hydrogels, supporting cell growth in diverse tissues. These properties have enabled their successful use in scaffolds for cartilage, bone, intervertebral disc, and cardiac regeneration, highlighting their potential as next-generation biomaterials for both load-bearing and electroactive applications.

#### 3.2.2. Wound Dressing

Cutaneous wound healing is a highly dynamic and coordinated biological process involving four overlapping stages: hemostasis, inflammation, proliferation, and tissue remodeling [80]. However, bacterial infection remains one of the most significant challenges that can disrupt this healing cascade, leading to delayed recovery and increased risk of complications [81]. Therefore, effective wound management requires dressings that not only protect the wound but also actively support the healing process. An ideal wound dressing should meet several key criteria: it must be non-toxic, non-allergenic, and capable of maintaining a moist environment at the wound site. It should allow gas exchange, absorb excess exudate and toxins, prevent bacterial colonization, and ideally possess intrinsic antimicrobial properties. Additionally, the dressing should be easy to remove without causing further trauma to the wound bed [81,82,83].

In recent years, CNF-based hydrogels have emerged as promising candidates for advanced wound dressings due to their biocompatibility, high water-holding capacity, structural similarity to the extracellular matrix, and potential for functionalization with antimicrobial or therapeutic agents (Table 2). For instance, Li et al. developed a CNF-based hydrogel composed of chitosan (CS), CNF, and tannic acid (TA), demonstrating strong antibacterial activity and hemostatic performance for treating drug-resistant bacterial infections [77]. In this system, CNFs serve dual roles as both structural and functional elements. Structurally, they form a nanofibrous three-dimensional network that reinforces the hydrogel matrix, mimics the ECM, and supports cellular adhesion and proliferation (Figure 5B, top). Functionally, their abundant hydroxyl groups facilitate strong hydrogen bonding with TA, enabling stable incorporation and enhancing antibacterial efficacy through membrane disruption and protein denaturation (Figure 5B, bottom). Therefore, CNFs significantly contribute to the hydrogel’s mechanical robustness, bioactivity, and antimicrobial functionality, making it highly effective for the treatment of infected wounds. In another study, TA was also utilized as an antibacterial and antioxidant agent in a glucose-responsive hydrogel composed of CNFs, 3-acrylamidophenyl boronic acid (AAPBA), and acrylamide (AM) [84].

In a related development, Zhang et al. developed a hybrid hydrogel of CNF, polyvinyl alcohol (PVA), and curcumin-modified silver nanoparticles (cAg) through a one-step synthesis method based on hydrogen bonds, dynamic boronic ester bonds, and coordinate covalent bonds [87]. In this hydrogel system, CNFs play a vital dual role [87]. Firstly, CNFs act as a structural matrix for anchoring and uniformly dispersing cAg, which endow the hydrogel with broad-spectrum antibacterial activity against pathogens such as *S. aureus*, *E. coli*, and *C. albicans*. The high surface area and reactive hydroxyl groups on CNFs facilitate the formation of coordinate interactions with cAg, improving nanoparticle stability, preventing aggregation, and enabling sustained release. Secondly, the intrinsic self-assembly of CNF chains into nanoscale interpenetrating hierarchical networks enhances the hydrogel’s mechanical performance, flexibility, self-healing, and tissue adhesion. These effects are attributed to the synergistic interplay of physical interaction, such as hydrogen bonding and polymer chain entanglement, and dynamic covalent borate ester bonding between CNFs and PVA chains. Moreover, the reactive surface chemistry of CNFs further supports the hydrogel’s multifunctionality, including its biodegradability and biocompatibility, ultimately promoting re-epithelialization, collagen deposition, and accelerated wound healing.

Overall, CNF-based hydrogels are promising wound dressing materials, combining biocompatibility, high water retention, and ECM-like architecture to support tissue repair. Their surface chemistry allows functionalization with bioactive agents, enabling antibacterial, antioxidant, hemostatic, and self-healing properties. These features enhance mechanical stability, reduce infection risk, and promote faster, more effective healing.

#### 3.2.3. Three-Dimensional Cell Culture Platforms

Three-dimensional (3D) cell culture systems better replicate the architecture, microenvironment, and cell–cell interactions of native tissues than conventional two-dimensional cultures, making them valuable for tissue engineering, disease modeling, and drug screening. Hydrogels are ideal 3D culture matrices due to their biocompatibility, tunable properties, and high water content [89]. CNF-based hydrogels offer additional advantages, including ECM-like nanofibrous architecture, adjustable mechanical strength and porosity, and the potential for biofunctionalization to support cell adhesion, proliferation, and differentiation [42].

CNF-based hydrogels formed either by self-assembly or ionic crosslinking have been have shown strong potential as 3D cell culture matrices for stem cells and organoids (Table 3). In the case of self-assembled CNF hydrogels without added crosslinkers, Sanandiya et al. demonstrated that injectable, surface-oxidized CNF thixogels supported high viability, long-term survival, and spherical morphology of human breast cancer cells and mouse embryonic stem cells [90]. These hydrogels exhibited shear-thinning and thixotropic behavior, remaining flowable under applied stress and rapidly recovering their structure within 60 s, while forming 3D networks through flexible nanofiber entanglement. The mechanical moduli could be tuned by adjusting CNF concentration or applying external stimuli. Similarly, TEMPO-oxidized CNF hydrogels with shear-healing and shear-thinning properties were shown to effectively support hepatic differentiation of human liver organoids into functional hepatocyte-like cells [78]. These organoids exhibited comparable expressions of hepatic genes, hepatocyte functions, and polarization to those cultured in Matrigel, suggesting that CNF-based hydrogels could serve as a promising, biocompatible alternative to Matrigel for tissue engineering and regenerative medicine applications (Figure 5C).

In ionic crosslinking systems, the introduction of carboxyl groups into CNFs via TEMPO oxidation enables the formation of metal–carboxylate complexes with multivalent cations, improving both biochemical and mechanical properties. Kim et al. reported the successful 3D encapsulation of osteoblasts in Ca^2+^-crosslinked CNF hydrogels, achieving high cell viability and long-term proliferation [91]. Notably, the cells remained uniformly distributed throughout the CNF hydrogel, avoiding the cell sinking observed in Matrigel. To further enhance biofunctionality, Curvello et al. functionalized TEMPO-oxidized (TON) and TEMPO/periodate-oxidized (TPON) CNFs with RGD peptides to promote cell attachment and differentiation [79]. Both TON and TPON hydrogels could be crosslinked with either Mg^2+^ or Ca^2+^ ions for intestinal organoid culture (Figure 5D). Due to their higher carboxyl content, TPON fibers allowed greater RGD grafting, providing stronger biochemical cues for cell adhesion. Organoids cultured in these hydrogels maintained viable regions for up to four days, and cell clusters recovered from Mg^2+^-crosslinked hydrogels could be passaged with intact RNA.

**Table 3:** CNF-based hydrogel as 3D cell culture platforms.

Hydrogel Composition/Type	Formation Method	Functionalization	Cell Type	References
TEMPO-oxidized CNF/injectable thixogel	Self-assembly (without added crosslinker)	**-**	Human breast cancer (MCF-7) and mouse embryonic stem cells (mESC; E14TG2A)	[90]
TEMPO-oxidized CNF/Injectable thixogel	Self-assembly (without added crosslinker)	**-**	Human liver organoid	[78]
TEMPO-oxidized nanofibrillated cellulose (NFC)/bulk gel	Self-assembly (without added crosslinker)	**-**	Mesenchymal stem cells (MSCs)	[92]
TEMPO-oxidized CNF/bulk gel	Calcium ion (Ca^2+^) crosslinking	**-**	Pre-osteoblast cells (MC3T3-E1)	[91]
TEMPO-oxidized CNF/bulk gel	Calcium ion (Ca^2+^) crosslinking	Fibronectin-derived moieties (RGD peptides), laminin-1, insulin-like growth factor (IGF-1)	Small intestinal organoids	[93]
TEMPO-oxidized (TON) and TEMPO/periodate-oxidized (TPON) CNF/bulk gel	Magnesium (Mg^2+^) and calcium ion (Ca^2+^) crosslinking	Fibronectin-derived moieties (RGD peptides)	Intestinal organoids	[79]
TEMPO-oxidized CNF/microgel	Calcium ion (Ca^2+^) crosslinking	Hyaluronic acid (HA)	Human adipose-derived stem cell (hADSC)	[94]

Moving beyond bulk hydrogels, Goh et al. demonstrated the fabrication of TEMPO-oxidized CNF hydrogel microbeads for human adipose-derived stem cell (hADSC) culture by simply dropping cell-containing gel precursors into a CaCl_2_ solution [94]. To improve long-term culture performance, hyaluronic acid (HA) was incorporated into the CNF microbead matrix via physical interactions. Microbeads containing 0.2% high-molecular-weight HA (700 kDa) significantly enhanced cell proliferation, VEGF secretion, and stemness maintenance compared to bare CNF microbeads. This HA-incorporated CNF microbead system thus offers a low-cost, sustainable microgel platform suitable for stem cell 3D culture.

CNF-based hydrogels, whether self-assembled or ionically crosslinked, provide tunable, ECM-mimicking 3D culture environments that support high cell viability, proliferation, and differentiation. They perform comparably to Matrigel while avoiding its key drawbacks, namely its animal origin, variability, undefined composition, and high cost, offering a sustainable, reproducible, and customizable alternative for advanced stem cell and organoid culture systems.

## 4. Summary and Outlook

Over the past decades, biomedical materials have advanced substantially in terms of CNF hydrogel research and development. These advanced materials possess intricate three-dimensional networks that can retain substantial amounts of water while maintaining robust structural integrity. This unique combination of properties has established CNF hydrogels as promising candidates across multiple biomedical domains, including cellular research, regenerative medicine, and therapeutic delivery systems.

Current research has revealed several areas that require further investigation before the widespread implementation of these hydrogels becomes feasible. In cellular applications, a major concern centers on the methodology used for hydrogel dissolution post-culture. Recent studies rely heavily on enzymatic breakdown processes; however, insufficient research exists on the extended impact of these enzymes on cellular health and function. The scope of cellular research using CNF hydrogels has remained relatively narrow, focusing predominantly on specific cell lines while leaving vast areas of potential applications, such as complex human organ culture systems, largely unexplored. The field of regenerative medicine presents additional limitations, particularly in terms of optimizing bioprinting processes. Current evidence suggests that pure CNF systems rarely achieve optimal printing outcomes without requiring supplementary biomaterials to be incorporated. Additionally, applications in wound management typically require antimicrobial agents to be integrated to achieve the desired therapeutic outcomes.

Initial medical trials have generated encouraging results in terms of biocompatibility. Notable research involving burn treatment applications yielded favorable safety profiles with the minimal occurrence of adverse reactions, i.e., clinical trials [95] showed no allergic reaction or inflammatory response to CNF wound dressings. These preliminary findings, though promising, highlight the critical need for further comprehensive safety evaluations. The toxicology assessment and long-term evaluation of in vivo toxicity and biocompatibility of CNF-based hydrogels remain a crucial issue for real clinical applications [96]. The development of standardized assessment protocols remains essential for advancing medical implementation.

The transition to commercial-scale production introduces additional complexities. A critical challenge involves understanding and controlling material variability owing to different source materials and processing methods. Although some studies have investigated the effects of aspect ratio, surface charge, and fabrication conditions on CNF properties [97,98,99], maintaining consistent quality under high production volumes remains challenging. Advanced manufacturing technologies, particularly in the realm of precision printing, show promise in terms of addressing some of these challenges by forming precisely controlled structures. However, these processes need to be optimized for large-scale implementation, and therapeutic delivery systems could benefit significantly from the adaptable nature of CNF hydrogels, particularly for developing responsive release mechanisms. The potential for enhancing diagnostic capabilities through the precise control of surface chemistry represents another promising avenue for investigation. Additionally, the development of comprehensive testing standards will be crucial for regulatory compliance across various applications.

Moreover, the technological advancements in processing methods are promising. Recent innovations in manufacturing techniques have demonstrated potential for generating sophisticated structural arrangements while maintaining material integrity. These developments could yield efficient production methods while enabling excellent control over final product characteristics. Integration with existing medical technologies represents another critical area for development, requiring careful consideration of practical aspects such as sterilization procedures and storage requirements.

The environmental and economic advantages of using CNF hydrogels position them favorably for future development. The renewable nature and potential for cost-effective production of these hydrogels align well with current sustainability initiatives. However, realizing this potential will require continued advancement in processing efficiency and scale-up capabilities. The successful translation of laboratory findings to practical applications will depend on addressing the aforementioned challenges while maintaining the fundamental properties that render these materials attractive for biomedical applications.

Future research directions should focus on developing sophisticated controlled-release mechanisms, improving drug loading efficiencies, and addressing the challenges of large-scale production. Integrating multiple stimuli-responsive elements and developing precise targeting mechanisms represent promising areas for investigation. Research efforts should clarify the long-term stability and degradation behavior of CNF hydrogels under physiological conditions and the interactions with various therapeutic agents. The cost-effectiveness and sustainability of CNF enhance its attractiveness from the environmental and financial perspectives, rendering it a promising material for next-generation drug delivery systems.

## Figures and Tables

**Table 1:** CNF-based hydrogel as scaffold materials in tissue engineering applications.

Hydrogel Composition/Type	Formation Method	Tissue Type	Role of CNF	Key Properties/Application	References
Poly(vinyl alcohol) (PVA), CNF	γ-ray irradiation, annealing, and rehydration	Cartilage	Reinforced mechanical properties via hydrogen bonding and molecular alignment with PVA matrix.	High tensile strength Good lubrication Mechanical mimicry of cartilage Suitable for cartilage and load-bearing tissue regeneration	[35]
TEMPO-oxidized cellulose nanofiber (TOCN), Decellularized extracellular matrix (dECM), Sodium alginate (SA)/3D printable	Two-step calcium ion (Ca^2+^) crosslinking		Improved viscoelastic behavior, stability, mechanical properties as well as printability of the scaffolds. Improved cell penetration in the scaffold.	Good printability High cell proliferation, penetration, and chondrogenic differentiation Suitable for cartilage tissue regeneration	[64]
Oxidized alginate (OSA), Gelatin (Gel), and CNF/Injectable and self-healing	One-step Schiff base reaction (aldehyde–amine)	Bone	Reinforced mechanical properties via hydrogen bonding. Rheological modifiers contributed to the shear-thinning behavior, facilitating injectability. Induced biomineralization via Ca^2+^ chelation.	Osteoconductive Sufficient mechanical strength Suitable for bone tissue repair	[65]
Enzymatically mineralized TEMPO-oxidized bacterial cellulose nanofibers (m-TOBC), Mesoporous silica nanoparticles (MSNs) loaded with the angiogenic drug dimethyloxalylglycine (DMOG), Gelatin methacryloyl (GelMA)/3D printable	Visible light (405 nm) crosslinking after 3D printing		Improved the printability of GelMA ink. Improved the mechanical properties of the hydrogel. Improved the osteo-conduction of the hydrogel.	Osteoconductive and creates an angiogenic microenvironment for enhancing bone repair Suitable for bone tissue repair	[66]
Viscous chitosan (CHI), CNF/Injectable	Physical mixing, no crosslinker (rheology-based structuring)	Intervertebral Disc (IVD)	Improved the post-injection stability of the hydrogel via hydrogen bonding and hydrophobic interaction with CHI. Improved hydrogel elastic modulus, mimicking native disc stiffness.	Restores disc height and biomechanics (in vivo) Suitable as non-cellularized, bioactive materials for disc repair	[67]
Viscous chitosan (CHI), CNF	Physical mixing and further neutralized with sodium hydroxide (NaOH)		Improved the mechanical properties of CHI hydrogel.	Restores disc biomechanics (in vivo) Serves as contention patches against nucleus protrusion Supports cell growth in a 3D environment Suitable for IVD tissue engineering	[68]
Poly (ethylene oxide)-poly(propylene oxide)-poly(ethylene oxide) block copolymer-diacrylate (PEO-PPO-PEO-DA), CNF, Gelatin methacryloyl (GelMA)/Injectable	UV crosslinking (365 nm at 6 mW/cm^2^)	Meniscus	Improved the mechanical characteristics (compression strength, compression modulus, ultimate tensile strength, tensile modulus, and elongation at break) of injectable hydrogels.	Good physicochemical properties Promoted cell viability Suitable and potential for meniscus tissue engineering	[69]
Poly (vinyl alcohol) (PVA), Glycidyl Methacrylate (GMA), CNF/Injectable	UV crosslinking (365 nm at 6 mW/cm^2^)		Improved the mechanical characteristics (compression strength and modulus) of injectable hydrogels. Improved human cartilage stem/progenitor cells (CSPCs) proliferation.	Good physicochemical properties Promoted cell proliferation Potential for meniscus tissue engineering	[70]
Pluronic^®^ F-127 (PEO99–PPO65–PEO99), Chitosan, Gold nanoparticles attached on TEMPO-oxidized bacterial cellulose nanofibers (Au@OBC)	Physical gelation via thermo-responsive behavior	Cardiac	Acted as stabilizing and dispersing agents for gold nanoparticles (AuNPs), ensuring their uniform distribution within the hydrogel matrix. This homogeneous dispersion enhances both the mechanical strength and electroconductivity of the hydrogel.	Thermosensitive Electroconductive Good physicochemical properties Potential to be employed as a platform for electroactive tissue repair	[71]
TEMPO-oxidized cellulose nanofibers (TOCN), Pyrrole monomer (PPy)	Iron ion (Fe^3+^) crosslinking followed by in situ polymerization of PPy in the presence of Fe^3+^		Provided mechanical reinforcement via a nanofibrous network. Served as a template for uniform dispersion of PPy monomers, enabled homogeneous in situ polymerization of PPy.	Good mechanical properties and conductive scaffold Promotes cell growth and proliferation as well as myocardial-specific protein expression Potential scaffold material for cardiac tissue engineering	[72]
Porcine skin collagen, TEMPO-oxidized cellulose nanofibers (CNF), Dexamethasone	First linking collagen and CNFs via carbodiimide chemistry, followed by photo crosslinking	Corneal	Supported the double-crosslinked hydrogel network. Enhanced mechanical strength of the hydrogel through integration into both crosslinking phases.	Good mechanical strength with sufficient transparency Sustained anti-inflammatory activity Potential as corneal implant to treat corneal stromal disease	[73]
TEMPO-oxidized cellulose nanofibers (CNF), Telechelic difunctional PEG (DF-PEG), Glycol chitosan (CS)/Injectable and self-healing	Schiff base reaction between aldehyde-functionalized telechelic difunctional PEG (DF-PEG-CHO) and amino groups on chitosan (CS)	Neural	Enhanced self-healing of hydrogel via strain-sensitive reinforcement. Improved thermal stability of the hydrogel. Prolonged biodegradation by stabilizing the network.	Tunable self-healing Enhanced neural stem cell oxygen metabolism and neural differentiation, and neuroregeneration (in vitro and in vivo) Potential as scaffold in neural regeneration	[74]
Fragmented short-length TEMPO-oxidized bacterial cellulose nanofibers (sOBC), Gelatin methacryloyl (GelMA), Transforming growth factor beta (TGF-β), and Fibroblast growth factor (FGF)	UV crosslinking	Tracheal	Serves as the bioactive surface for TGF-β loading. Enhanced structural stability of the hydrogel and mechanical strength via hydrogen bonding.	Controlled and sustained release of growth factors Regeneration of mature neo-cartilage with typical lacunae, supports cartilage regeneration under low density cells Formation of cartilage-ring analog Potential candidate for tracheal defects repair	[75]

**Table 2:** CNF-based hydrogel as wound dressing.

Hydrogel Composition/Type	Formation Method	Antimicrobial or Therapeutic Agents	Role of CNF	Key Findings	References
Chitosan (CS), CNF, Tannic acid (TA)	Both chemical (amino group of the CS chain and the ester group of genipin) and physical crosslinking (hydrophobic and hydrogen bond formed between CS, CNF, and TA)	Tannic acid (TA)	Reinforced the hydrogel matrix, mimics the ECM, and supports cellular adhesion and proliferation Enabled stable incorporation of TA via hydrogen bonding to enhance antibacterial efficacy	Exhibited extraordinary hemostatic ability during the bleeding phase of the wound. Accelerated cell proliferation and differentiation. Suppressed bacterial growth, facilitating wound microenvironmental cleaning, promoting hair follicle and vessel regeneration, and expediting wound healing on full thickness rat skin wound.	[77]
CNFs, Tannin (TA), 3-acrylamidophenyl boronic acid (AAPBA), Acrylamide (AM)/Self-healing and glucose responsiveness	AAPBA was copolymerized with AM using APS and MBA as crosslinker, CNFs and TA were crosslinked with poly(AM– AAPBA) through the formation of dynamic borate ester bonds between the boronic acid groups in AAPBA and the *o*-dihydroxy groups in TA and CNFs	Tannin (TA)	Improved the tensile stress of composite hydrogels Endowed the hydrogel with self-healing property through the dynamic borate ester bonding with AAPBA and TA	Released more tannin in glucose solution. Exhibited high antioxidant activity. Exhibited antimicrobial effect against *E. coli* and *S. aureus*. Able to adsorb proteins (bovine serum albumin (BSA)).	[84]
Poly(vinyl alcohol) (PVA), Borax, Dopamine-grafted oxidized carboxymethyl cellulose (OCMC-DA), CNF, Neomycin (NEO)/Self-healing and pH responsive	Dynamic reversible borate ester linkages and hydrogen bonds between OCMC-DA, PVA, and CNF, along with dynamic crosslinking imine linkages between NEO and OCMC-DA	Neomycin (NEO)	Reinforced the hydrogel matrix, enabling the integration of excellent ductility, self-adaptability, biodegradability, and biocompatibility	Exhibited excellent self-healing ability and stretchability (3300%). Exhibited antibacterial effect against a broad spectrum of bacteria (*E. coli* and *S. aureus*).	[85]
Poly(vinyl alcohol) (PVA), Borax, Resveratrol-grafted cellulose nanofibrils (RPC)/Self-healing and pH-responsive	Dynamic reversible borate ester linkages and hydrogel bond between PVA, borax and RPC	Resveratrol	Enhanced the mechanical strength of the hydrogel by promoting extra hydrogen bonds and physical entanglement	Exhibited robust mechanical properties (fracture strength of 149.6 kPa), high self-healing efficiency (>90%), and excellent adhesion performance (tissue shear stress of 54.2 kPa). Showed pH-responsive drug release behavior, the cumulative release amount of resveratrol in pH 5.4 was 2.33 times that of pH 7.4. Exhibited excellent antioxidant effects and antibacterial effect against *S. aureus*. Exhibited excellent antibacterial effect, skin tissue regeneration, and wound closure capabilities on the skin wounds of mice infected with *S. aureus*.	[86]
CNF, polyvinyl alcohol (PVA), and curcumin-modified silver nanoparticles (cAg), Borax	One-step polymerization (hydrogen bonds between CNF and PVA, dynamic boronic ester bonds between borate ion, CNF and PVA, and coordinate covalent bonds between Ag and CNF)	Curcumin-modified silver nanoparticles (cAg)	Acted as a structural matrix for anchoring and uniformly dispersing cAg, which endows the hydrogel with broad-spectrum antibacterial activity Enhanced the hydrogel’s mechanical performance, flexibility, self-healing, and tissue adhesion via intrinsic self-assembly of CNF chains into nanoscale interpenetrating hierarchical networks	Exhibited combined superiorities of excellent mechanical performances (tensile stress of 231 kPa and compressive stress of 1.23 MPa), self-healing efficiency of 94.55%, and favorable adhesion strength of 48 kPa. Exhibited robust antimicrobial activity against *S. aureus*, *E. coli*, and *C. albicans* and antioxidant activity. Displayed outstanding drug release behavior of Ag+ and curcumin, great swelling ratio (>700%), enhanced cell viability (over 98%) and proliferation of L929 cells. Showed good in vivo wound closure (wound closure rate up to 97%) collagen deposition and granulation thickness.	[87]
Dopamine-modified Tempo-oxidized cellulose nanofibers (DA-TCNF), Chitosan, (3-aminobenzeneboronic acid)- grafted oxidized dextran (POD), and Poly(vinyl alcohol) (PVA)/ROS and pH responsiveness	POD and DA-TCNF form dynamic Schiff base, boronic ester linkages and hydrogen bond with PVA and chitosan	Mangiferin and Vitamin C	Dopamine-modified TCNF introduced catechol groups into the hydrogel, mimicking the strong adhesive properties of mussel proteins and enabling firm skin attachment Contributed to mechanical stability and dynamic responsiveness of the hydrogel	Exhibited self-healing, superior mechanical strength, self-adhesion, 3D printability, injectability, and hemostatic properties. Exhibited excellent antioxidant and antibacterial activity against *S. aureus* and *E. coli*. Exhibited dual pH- and ROS-responsive controlled release of mangiferin and vitamin C. Improved wound healing of full thickness rat wound skin by facilitating angiogenesis, collagen deposition, and the inhibition of inflammation and bacterial infections.	[88]

## Data Availability

No new data were created or analyzed in this study.

## Abbreviations

The following abbreviations are used in this manuscript:

CNF	Cellulose nanofibril
CNC	Cellulose nanocrystal
TEMPO	2,2,6,6-tetramethyl-1-piperidinyloxy
SEM	Scanning electron microscope
U-CNF	Untreated CNF
CM-CNF	Carboxymethylated CNF
Q-CNF	Quaternized CNF
PVP	Polyvinylpyrrolidone
PEG	Poly (ethylene glycol)
PNIPAm	Poly(N-isopropylacrylamide)
SA	Sodium alginate
TH	Tetracycline hydrochloride
MPDA	Mesoporous polydopamine
GO	Graphene oxide
TOCN	TEMPO-oxidized CNF
PPy	Polypyrrole
